# Association of preconception premenstrual disorders with perinatal depression: an analysis of the perinatal clinical database of a single Japanese institution

**DOI:** 10.1186/s13030-024-00323-7

**Published:** 2024-12-23

**Authors:** Takuma Ohsuga, Miho Egawa, Kaori Tsuyuki, Akihiko Ueda, Maya Komatsu, Yoshitsugu Chigusa, Haruta Mogami, Masaki Mandai

**Affiliations:** Department of Gynecology and Obstetrics, Kyoto University Graduate School of Medicine, 54 Shogoin-Kawahara-Cho, Sakyo-Ku, Kyoto, 606-8507 Japan

**Keywords:** Perinatal mental health, Postpartum depression, Prenatal depression, Premenstrual disorders, Premenstrual dysphoric disorder, Premenstrual syndrome, Preconception

## Abstract

**Background:**

Recent studies have identified premenstrual disorders (PMDs) as a risk factor for postpartum depression. However, routine screening for preconception PMDs is not yet common in Japan. This study investigated the association between preconception PMDs and perinatal depression in a single tertiary care setting.

**Methods:**

We analyzed data from pregnant women who gave birth at Kyoto University Hospital between April 2020 and October 2023. The Premenstrual Symptoms Screening Tool was administered at the first postconception visit to retrospectively assess PMD status before the current pregnancy. The Edinburgh Postnatal Depression Scale (EPDS) was administered during pregnancy and one month postpartum as a prospective measure of perinatal depression. EPDS cutoff values were set at 12/13 during pregnancy and 8/9 at one month postpartum.

**Results:**

Of the 781 women analyzed, 53 had preconception PMD. Univariate and multivariate logistic regression analyses revealed that preconception PMD was associated with an EPDS score of ≥ 13 during pregnancy, with a crude odds ratio (OR) of 5.78 (95% confidence interval [CI]: 2.70–11.75) and an adjusted OR of 3.71 (95% CI: 1.54–8.35). For an EPDS score of ≥ 9 at 1 month postpartum, the crude OR was 3.36 (95% CI: 1.79–6.12) and the adjusted OR was 2.16 (95% CI: 1.04–4.35).

**Conclusions:**

Our findings indicate that preconception PMDs are a significant risk factor for both depression during pregnancy and postpartum depression. These results support the implementation of preconception PMD screening during antenatal checkups as a preventive measure and to identify women in need of early mental health care.

## Background

Perinatal depression, as defined by the Diagnostic and Statistical Manual of Mental Disorders, Fifth Edition (DSM-5), refers to a major depressive episode occurring during pregnancy or within the first four weeks postpartum [1]. It is a significant public health concern due to its high prevalence and its detrimental effects on mothers, infants, and families. A 2017 systematic review and meta-analysis reported an overall pooled prevalence of perinatal depression at 11.9% (95% confidence interval [CI]: 11.4–12.5) [2]. Depression during pregnancy is linked to increased risks of preterm delivery and low birth weight [3]. Additionally, perinatal depression is associated with poor maternal bonding, child maltreatment, maternal suicide, and adverse developmental outcomes in children, including deficits in social–emotional, neurocognitive, language, motor, and adaptive behaviors [4, 5]. Symptoms of depression during pregnancy also predict postpartum depression [6], with some cases beginning prior to delivery [7]. Early identification and intervention for at-risk pregnant women are crucial for preventing perinatal depression [8].

Premenstrual syndrome (PMS) encompasses a range of physical and emotional symptoms that occur during the luteal phase before menstruation and generally diminish once menstruation begins. The severity and pattern of these symptoms can vary widely among individuals. A more severe form of PMS, known as premenstrual dysphoric disorder (PMDD) [9], is recognized as a psychiatric disorder in the DSM-5 [1]. Both PMS and PMDD are often referred to collectively as premenstrual disorders (PMDs) [10–12]. Despite increasing public awareness of PMDs, many women with severe symptoms do not seek medical help. Recent studies have increasingly explored the link between PMDs and perinatal depression, primarily focusing on postpartum depression, with fewer addressing depression during pregnancy [13–16]. Although screening for PMDs during preconception could potentially facilitate early intervention for perinatal mental health issues, this practice is not yet widespread in Japan.

Prenatal and postpartum mental health issues are gaining increased recognition in society. However, clinical records often prioritize physical health information, with psychosocial and mental health data frequently being insufficient or fragmented. Since 2020, our institution has been using a comprehensive perinatal clinical database we developed that integrates psychosocial and mental health information to enable a more holistic approach to perinatal care. This database includes data from questionnaires on preconception PMDs as well as prenatal and postpartum depression. This study used the database to investigate the association between preconception PMDs and depression during pregnancy and postpartum.

## Methods

### Study design and participants

The perinatal clinical database of Kyoto University Hospital (a tertiary care facility) includes the data of all pregnant women treated at the hospital. A patient is registered in the database during the first visit postconception. The Edinburgh Postnatal Depression Scale (EPDS) was administered mid-pregnancy and one month postpartum. The Premenstrual Symptoms Screening Tool (PSST) was administered during the first visit postconception and used to assess the patient’s PMD status before conception. Information from the PSST and EPDS are included in the database. This study was conducted using the data of pregnant women who underwent live deliveries at the hospital between April 2020 and October 2023 after at least 22 weeks of gestation. Women were excluded if they had missing PSST data, had missing EPDS data during pregnancy and postpartum or were experiencing a second or subsequent pregnancy during the study period. The study protocol was reviewed and approved by the Ethics Committee of Kyoto University Graduate School and Faculty of Medicine (Approval No. R2262). Informed consent was obtained through an opt-out system, with participants receiving detailed information via disclosure documents.

### Measurements

#### Exposure: preconception PMDs

The Japanese version of the PSST was administered during the first visit postconception to assess premenstrual symptoms prior to the current pregnancy. The PSST, originally developed by Steiner et al. based on the PMDD criteria from the Diagnostic and Statistical Manual of Mental Disorders, Fourth Edition (DSM-IV) [17], is a widely used self-report tool for screening PMDs [18–22]. In this study, the PSST was translated into Japanese by the original developers and used with permission from the copyright holder. The PSST includes 14 premenstrual symptoms and five types of life interference caused by these symptoms, each rated on a 4-point Likert scale: “not at all,” “mild,” “moderate,” or “severe.” Based on the PSST results, participants were categorized as follows: those meeting DSM-IV criteria for PMDD were classified as having “PMDD”; those with clinically evident PMS symptoms but not meeting PMDD criteria were classified as having “moderate/severe PMS”; and all others were classified as having “no/mild PMS.” In this study, participants with “PMDD” or “moderate/severe PMS” were grouped together in a PMDs group, whereas the others were classified into a non-PMDs group. To maintain objectivity, the obstetricians and midwives involved in patient care were not informed of the PSST results.

### Outcomes: perinatal depression

The Japanese version of the EPDS was used to prospectively assess perinatal depression. The EPDS was administered during pregnancy, generally in the second trimester, and again at one month postpartum [23]. The EPDS is a widely used self-report questionnaire designed to screen for postpartum depression. It consists of 10 items rated on a 4-point scale (0–3), with total scores ranging from 0 to 30 [24]. In this study, a cutoff score of 8/9 was used for assessing postpartum depression. The EPDS has demonstrated validity and reliability in Japanese populations [23]. Although there is no universally established cutoff for the EPDS during pregnancy in Japan, this study used a cutoff value of 12/13 based on a previous study [25].

### Statistical analysis

First, to show the characteristics of the PMDs and non-PMDs groups, continuous variables are presented as means with standard deviations and categorical variables are presented as frequencies and percentages. We then analyzed the association between PMDs during preconception and depression during pregnancy and one month postpartum using univariate and multivariate logistic regression. The adjustment factors included age (continuous variable), pre-pregnancy body mass index (categorized as < 18.5, 18.5–25, or ≥ 25), parity (nullipara or multipara), the mother’s educational level (junior high school/high school or higher), history of psychiatric or psychosomatic consultations (yes or no), and issues such as relationship problems with a partner, financial difficulties, violence, or unemployment (yes or no). The number of adjustment factors was based on the one-in-ten rule [26]. All analyses were performed using R version 4.3.1.

## Results

**Fig. 1:**
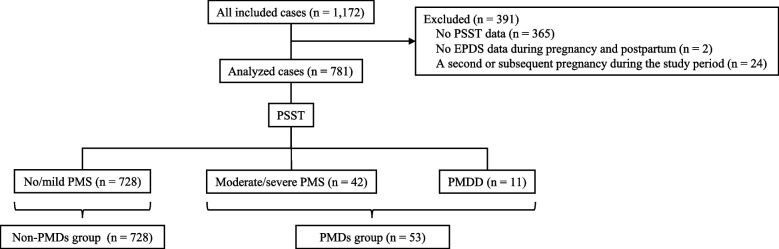
Flowchart of patient inclusion. Abbreviations: EPDS, Edinburgh Postnatal Depression Scale; PMDD, premenstrual dysphoric disorder; PMDs, premenstrual disorders; PMS, premenstrual syndrome; PSST, Premenstrual Symptoms Screening Tool

Of the 1,172 women treated during the study period, 781 were included in the analysis. We excluded 365 women with missing PSST data, 2 with missing EPDS data during pregnancy and postpartum, and 24 with a second or subsequent pregnancy during the study period. The non-PMDs group comprised 728 women classified as having “no/mild PMS” based on the PSST, whereas the PMDs group included 53 women: 42 with “moderate/severe PMS” and 11 with “PMDD” (Fig. 1). The characteristics of each group are detailed in Table 1. The PMDs group was more likely to have a history of psychiatric or psychosomatic consultations and to experience problems related to partner relationships, finances, violence, or unemployment.

**Table 1:** Basic characteristics of the PMDs and non-PMDs groups

	PMDs group (*n* = 53)	Non-PMDs group (*n* = 728)	*P* value*
Age at delivery, mean (SD) [years]	33.7 (4.5)	34.6 (5.2)	0.18
Parity, multipara, n (%)	24 (45.3%)	354 (48.6%)	0.71
Pre-pregnancy body mass index, mean (SD)	22.8 (5.0)	21.3 (3.4)	0.03
Mother's educational level			
Junior high school or high school, n (%)	18 (34.0%)	258 (35.4%)	0.88
Vocational school, junior college, university, or graduate school, n (%)	31 (58.5%)	405 (55.7%)	
History of psychiatric or psychosomatic consultation, n (%)	18 (34.0%)	92 (12.6%)	< 0.001
Infertility treatment, n (%)	21 (40.0%)	292 (40.1%)	1
Problems (relationship with partner, finances, violence, or unemployment), n (%)	7 (13.2%)	21 (2.9%)	< 0.001
Gestational week of delivery, mean (SD) [weeks]	37.7 (2.5)	37.7 (2.7)	0.91
Mode of delivery, cesarean section, n (%)	24 (45.3%)	370 (50.8%)	0.51
Weeks of gestaion responding to PSST, mean (SD)	17.3 (9.3)	20.1 (9.5)	0.03
Weeks of gestation responding to EPDS during pregnancy, mean (SD)	22.1 (6.3)	22.9 (6.8)	0.34

*Abbreviations*: *EPDS* Edinburgh Postnatal Depression Scale, *PMDs* Premenstrual disorders, *PSST* Premenstrual Symptoms Screening Tool, *SD* Standard deviation

^*^Welch's t-test or Pearson's Chi-squared test

The percentage of women with an EPDS score of ≥ 13 during pregnancy was 22.6% in the PMDs group compared with 4.7% in the non-PMDs group (Table 2). At one month postpartum, 34.0% of the women in the PMDs group had an EPDS score of ≥ 9, as opposed to 12.9% of the women in the non-PMDs group. Logistic regression analysis results are presented in Table 2. In univariate analysis, preconception PMDs were associated with an EPDS score of ≥ 13 during pregnancy, with a crude odds ratio (OR) of 5.78 (95% CI: 2.70–11.75). This association persisted after adjusting for age, history of psychiatric or psychosomatic consultations, and issues related to partner relationships, finances, violence, or unemployment, with an adjusted OR of 3.71 (95% CI: 1.54–8.35). Preconception PMDs were also associated with an EPDS score of ≥ 9 at one month postpartum in univariate analysis (crude OR: 3.36 [95% CI: 1.79–6.12]). This association remained significant after adjusting for age, psychiatric or psychosomatic consultations, problems related to partner relationships, finances, violence, or unemployment as well as maternal educational level and parity, with an adjusted OR of 2.17 (95% CI: 1.04–4.35).

**Table 2:** Perinatal depression assessed using EPDS in the PMDs and non-PMDs groups

	PMDs group (*n* = 53)	Non-PMDs group (*n* = 728)
During pregnancy		
EPDS total score		
Mean (SD)	8.9 (6.8)	4.3 (4.4)
≥ 13, n (%)	12 (22.6%)	34 (4.7%)
< 13, n (%)	41 (77.4%)	671 (92.2%)
Crude OR^a^ (95% CI)	5.78 (2.70 − 11.75)	ref
Adjusted OR^b^ (95% CI)	3.71 (1.54 − 8.35)	ref
At 1 month postpartum		
EPDS total score		
Mean (SD)	7.8 (6.4)	4.3 (4.0)
≥ 9, n (%)	18 (34.0%)	94 (12.9%)
< 9, n (%)	34 (64.2%)	596 (81.9%)
Crude OR^a^ (95%CI)	3.36 (1.79 − 6.12)	ref
Adjusted OR^c^ (95%CI)	2.17 (1.04 − 4.35)	ref

*Abbreviations*: *CI* Confidence interval, *EPDS* Edinburgh Postnatal Depression Scale, *OR* Odds ratio, *PMDs* Premenstrual disorders, *ref* Reference, *SD* Standard deviation

^a^Univariate logistic regression analysis

^b^Multivariate logistic regression analysis: adjusted for age, history of psychiatric or psychosomatic consultation, and problems (relationship with partner, finances, violence, or unemployment)

^c^Multivariate logistic regression analysis: adjusted for age, history of psychiatric or psychosomatic consultation, problems (relationship with partner, finances, violence, or unemployment), pre-pregnancy body mass index, maternal educational level, and parity

## Discussion

This prospective analysis of 781 pregnant women at a tertiary care hospital revealed that preconception PMDs are a significant risk factor for both depression during pregnancy and postpartum depression, with adjusted ORs of 3.71 (95% CI: 1.54–8.35) and 2.17 (95% CI: 1.04–4.35), respectively. These findings suggest that screening for preconception PMDs, along with assessing other risk factors for perinatal depression, is crucial for the early identification and management of women at risk. Early detection can facilitate timely mental health interventions and preventive measures. Although the diagnostic criteria for PMDs typically involve prospective symptom documentation, recall-based screening remains a practical approach, given that many women seek medical care only after becoming pregnant.

Increasing evidence suggests that preconception PMD is a significant risk factor for postpartum depression. In a systematic review and meta-analysis, Cao et al. demonstrated that pregnant women with a history of PMS had a higher risk of postpartum depression than those without PMS, with an OR of 2.20 (95% CI: 1.81–2.68) [13]. Additionally, Gastaldon et al. identified PMS as one of the most robust risk factors in an umbrella review of systematic reviews and meta-analyses on postpartum depression and depressive symptoms [27]. Our findings align with those of the aforementioned studies. However, there is limited research on the association between preconception PMDs and depression during pregnancy. Sugawara et al. conducted a prospective cohort study involving 1,329 Japanese pregnant women and found that those with premenstrual irritability before pregnancy had significantly higher scores on Zung’s Self-Rating Depression Scale throughout pregnancy and postpartum (at six time points: early pregnancy, mid-pregnancy, late pregnancy, 5 days postpartum, 1 month postpartum, and 6 months postpartum) compared with those without such irritability [16]. Similarly, Pataky et al. found in a study involving 687 pregnant women from German-speaking countries that PMS/PMDD, assessed using the PSST, was associated with increased odds of an EPDS score ≥ 10 during the perinatal period (late pregnancy, 1–2 weeks postpartum, and 4–6 weeks postpartum) [14]. Although our study used different scales and targeted a different population, these findings collectively support the notion that pre-pregnancy PMDs are also a risk factor for depression during pregnancy.

Our findings highlight the importance of managing PMDs even before conception. Addressing PMDs as an indicator of underlying issues and intervening with modifiable factors could potentially prevent various conditions, including perinatal depression. Both PMDs and perinatal depression involve complex pathophysiological mechanisms, with neuroendocrine, neurotransmitter, immune-inflammatory, and genetic factors contributing to their development. Although these mechanisms are not fully understood, there may be some overlap between the two conditions [28–30]. Epidemiologically, several common risk factors for PMDs and perinatal depression include nutritional deficiencies. Deficiencies of vitamin D, calcium, zinc, iron, and polyunsaturated fatty acids have been associated with these symptoms and may improve with supplementation [31–39]. Enhancing nutritional status prior to pregnancy may help reduce the risk of developing perinatal depression. Additionally, adversarial childhood experiences are a significant common risk factor for both PMDs and perinatal depression [40–44]. Providing strong social support for women with such experiences may mitigate their risk of developing these conditions.

## Limitations

This study has several limitations. First, both the EPDS and PSST are self-report screening tools, and the psychometric properties of the Japanese version of the PSST have not yet been thoroughly examined. Notably, recall bias may have influenced the study results: the participants were asked to recall their experiences with premenstrual symptoms before pregnancy. Further research is needed to determine the most effective method for screening PMDs before pregnancy during antenatal checkups. Additionally, establishing a specialized system for preconception medical examinations and enhancing educational efforts to encourage women with PMDs to seek medical care could improve diagnosis and management. Second, there was a significant amount of missing PSST data. The study was conducted in a manner that did not interfere with routine outpatient operations, which may have led to some data being overlooked or forgotten. Third, as the study was conducted at a tertiary care hospital, the findings may not be generalizable to other settings.

## Conclusions

In this study conducted at a tertiary care hospital, preconception PMDs were found to be associated with an increased risk of both depression during pregnancy and at one month postpartum, as measured by self-administered questionnaires. These findings underscore the importance of screening for preconception PMDs during antenatal checkups. Early detection through such screening can facilitate timely mental health interventions and preventive measures for pregnant women.

## Data Availability

The datasets analyzed during the current study are available from the corresponding author on reasonable request.

## Abbreviations

CI: Confidence interval
DSM-5: Diagnostic and statistical manual of mental disorders, Fifth Edition
DSM-IV: Diagnostic and statistical manual of mental disorders, Fourth Edition
EPDS: Edinburgh postnatal depression scale
OR: Odds ratio
PMDD: Premenstrual dysphoric disorder
PMDs: Premenstrual disorders
PMS: Premenstrual syndrome
PSST: Premenstrual symptoms screening tool
